# Recurrent mutations in NF-κB pathway components, *KMT2D,* and *NOTCH1/2* in ocular adnexal MALT-type marginal zone lymphomas

**DOI:** 10.18632/oncotarget.11548

**Published:** 2016-08-23

**Authors:** Patricia Johansson, Ludger Klein-Hitpass, Florian Grabellus, Georg Arnold, Wolfram Klapper, Roman Pförtner, Ulrich Dührsen, Anja Eckstein, Jan Dürig, Ralf Küppers

**Affiliations:** ^1^ Department of Haematology, University Hospital Essen, University of Duisburg-Essen, Essen, Germany; ^2^ Institute of Cell Biology (Cancer Research), Medical Faculty, University of Duisburg-Essen, Essen, Germany; ^3^ Institute of Pathology and Neuropathology, University Hospital Essen, West German Cancer Center, University Duisburg-Essen, Essen, Germany; ^4^ German Cancer Consortium (DKTK), Essen, Germany; ^5^ Center for Pathology Essen-Mitte, Essen, Germany; ^6^ Department of Pathology, Haematopathology Section and Lymph Node Registry, University Hospital Schleswig-Holstein, Campus Kiel/Christian-Albrecht University, Kiel, Germany; ^7^ Department of Oral and Cranio-Maxillofacial Surgery, Kliniken Essen-Mitte, Evang. Huyssens-Stiftung/Knappschaft GmbH, University Hospital of Essen, Essen, Germany; ^8^ Department of Ophthalmology, Molecular Ophthalmology Group, University of Duisburg-Essen, Essen, Germany; ^*^ Present address: Center for Pathology Essen-Mitte, Essen, Germany

**Keywords:** KMT2D, MALT lymphoma, NF-κB, NOTCH, ocular adnexal lymphoma

## Abstract

The pathogenesis of ocular adnexal marginal zone lymphomas of mucosa-associated lymphatic tissue-type (OAML) is still poorly understood. We analyzed 63 cases of such lymphomas for non-synonymous mutations in 24 candidate genes by amplicon sequencing. We validated frequent mutations in the NF-κB regulators MYD88, TNFAIP3 and TNIP1 in OAML, but also identified recurrent mutations in several additional components of the NF-κB pathway, including BCL10 and NFKBIA. Overall, 60% of cases had mutations in at least one component of NF-κB signaling, pointing to a central role of its genetic deregulation in OAML pathogenesis. Mutations in *NOTCH1* and *NOTCH2* were each found in 8% of cases, indicating a pathogenetic function of these factors in OAML. KMT2D was identified as the first epigenetic regulator with mutations in OAML, being mutated in 22% of cases. Mutations in *MYD88* were associated with an inferior disease-free survival. Overall, we identified here highly recurrent genetic lesions in components of the NF-κB pathway, of *NOTCH1* and *NOTCH2* as well as *KMT2D* in OAML and thereby provide major novel insights into the pathogenesis of this B cell malignancy.

## INTRODUCTION

Lymphomas of the ocular adnexa account for about 1-2% of Non-Hodgkin lymphomas (NHL) [1]. The most frequent type of primary ocular adnexal lymphomas are extranodal marginal zone lymphomas of the mucosa-associated lymphatic tissue (MALT) subtype (OAML), representing about 80% of such lymphomas [2–4]. OAML show a mature B cell phenotype and are derived from post-germinal center B cells [5, 6]. In some geographical areas, there is a link between OAML and chronic infection by Chlamydia psittaci, suggesting an etiological role of the infection in these instances [7, 8]. A number of recurrent chromosomal aberrations and genetic lesions have been identified for OAML. Trisomies of chromosome 3 and 18 are the most common chromosomal imbalances [9–11]. Mutually exclusive chromosomal translocations in OAML include t(1;14)(p22;q32) (*BCL10*/IgH), t(14;18)(q32;p21) (IgH/*MALT1*), t(11;18)(q21;q21) (*BIRC3*/*MALT1*), and t(3;14)(p14;q32) (*FOXP1*/IgH) [12–17]. BCL10 and MALT1 are positive regulators of NF-κB signaling, and FOXP1 also supports NF-κB activity, indicating a role of NF-κB deregulation in OAML pathogenesis. Although single studies reported frequencies of up to 20% of these translocations in OAML, the majority of investigations revealed that these four types of translocations are very rare in this type of lymphoma [12–17]. Regarding the role of NF-κB signaling in OAML, several further mechanisms of NF-κB activation in primary OAML were analyzed. TNFAIP3, a tumor suppressor and negative regulator of NF-κB [18], is inactivated by promoter methylation, mutations and/or deletions in about 15-30% of OAML [19–21]. Moreover, presumably activating mutations of *MYD88*, a factor physiologically linking toll-like receptor signaling to NF-κB activation, were found in about 6% of cases, and one analysis revealed recurrent *BCL10* mutations, whereas no or only very rare mutations were identified in *BIRC3, CARD11, CD79A, CD79B*, and *TNIP2* (*ABIN2*), further components of the NF-κB pathway [22, 23].

The frequent occurrence of genetic alterations of factors of the NF-κB signaling pathway in OAML prompted us to perform a targeted sequencing analysis for mutations in components of the NF-κB pathway. Our collection of 24 genes included a number of further genes which are known to be recurrently mutated in other B cell NHL (B-NHL), but which were not analyzed yet in OAML. This includes *NOTCH1*, *NOTCH2*, and *KMT2D*, which were recently identified as being frequently mutated in various types of low-grade B-NHL, as well as the tumor suppressor genes and main apoptosis regulators TP53 and FAS [24–31].

## RESULTS

### Detection of numerous recurrently mutated genes in OAML

In this study we analyzed primary OAML from 63 patients for mutations in 24 selected genes. The clinical characteristics of the patients are shown in Table 1. The median follow-up time was 62 months (range 19-194).

**Table 1:** Patients' characteristics*

Characteristics	No. of patients (n=63)	%
Age at diagnosis (years)		
Median	67	
Range	29–87	
Sex		
Male	40	63
Female	23	37
Ann-Arbor Stage at diagnosis		
I	37/46	80
II - IV	9/46	20
Localisation at diagnosis		
Orbita	20/46	43
Conjunctiva	21/46	46
Lacrimal gland	5/46	11
Treatment		
Radiotherapy	37/46	80
Immunochemotherapy	6/46	13
Anti-CD20 antibody	1/46	2
No therapy	2/46	4
Response to treatment (complete remission)		
Radiotherapy	35/37	95
Immunochemotherapy	1/5	20
Anti-CD20 antibody	0/1	0
Relapse	20/46	44
Death	4	6
Observation period (months)		
Median	62	
Range	19–194	

* Listed are only those patients for whom the complete set of clinical data is available.

The 24 genes included in the targeted sequencing analysis (Table 2) were selected based on their role in the NF-κB pathway and/or their known pathogenetic role in other types of B cell lymphomas. All coding exons of the genes were amplified. The mean sequence coverage across all samples was 160, with a range from 20 to 204. 98% of positions were covered at least 20 times. To exclude rare subclonal mutations and avoid problems with PCR errors, we considered only those positions that were covered by at least 100 reads (based on our own validation analysis, see below), and variants that accounted for at least 5% of the sequence reads at a given position. Analysis of the sequence data revealed non-synonymous mutations in 16 of the 24 genes analyzed, and only such non-synonymous mutations are considered in the following (Supplementary Table S1). No non-synonymous mutations were observed in *CD79A*, *FAS*, *IKBKG*, *MALT1*, *RIPK1***, *RIPK2*, *TNIP2* and *TRAF3*. Some lymphomas exhibited two mutations in *KMT2D* or *TNFAIP3*. For 16 patients no mutations were observed. The average number of mutated genes per lymphoma was 1.3 (range 0-4). The non-synonymous mutations consisted of 70% substitutions, 20% deletions, 9% insertions, and 1% complex variations (combinations of exchanges and/or insertions/deletions). Sixty-four percent of the non-synonymous mutations were either nonsense mutations or deletions/insertions generating a frameshift, or were replacement mutations that we scored as likely damaging by at least three of five evaluation tools applied (Supplementary Table S1), indicating that a high fraction of non-synonymous mutations are functionally relevant.

**Table 2:** Genes included in the sequence analysis

Gene	Signaling pathway
BCL10	NF-κB canonical
BIRC3 (API2)	NF-κB canonical and non-canonical
CARD11	NF-κB canonical
CD79A	NF-κB canonical
CD79B	NF-κB canonical
CYLD	NF-κB canonical and non-canonical
FAS (TNFRSF6)	Extrinsic apoptosis
IKBKG (NEMO)	NF-κB canonical
KMT2D (MLL2)	Methylation
MALT1	NF-κB canonical
MAP3K7 (TAK1)	NF-κB canonical
MAP3K14 (NIK)	NF-κB non-canonical
MYD88	NF-κB canonical
NFKBIA	NF-κB canonical
NOTCH1	Notch
NOTCH2	Notch
RIPK1	NF-κB canonical
RIPK2	NF-κB canonical
TNFAIP3	NF-κB canonical
TNIP1 (ABIN1)	NF-κB canonical
TNIP2 (ABIN2)	NF-κB canonical
TP53	Extrinsic apoptosis
TRAF3	NF-κB non-canonical
TRAF6	NF-κB canonical

**Figure 1:**
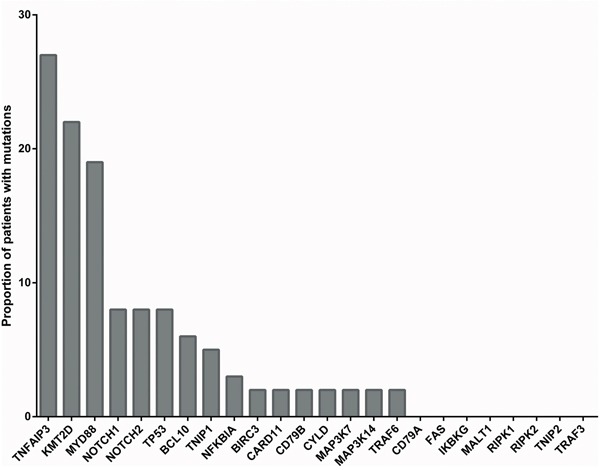
Proportion of OAML cases with mutations in the 24 genes analyzed Given is the proportion of the 63 OAML patients included in the analysis that carried mutations in the 24 genes analyzed.

Regarding components of NF-κB signaling, the most frequently mutated genes in our patient cohort were *TNFAIP3* (27%), *MYD88* (19%), and BCL10 (6%) (Figure 1). We observed mutations in several genes of the canonical NF-κB signaling pathway, but only one mutation was seen in one of the two key factors of the alternative NF-κB pathway included in the analysis, i.e. MAP3K14.

Mutations in *MYD88* (Leu265Pro) have been previously described as somatic oncogenically active mutations in several lymphoma entities [22, 32, 33]. The pattern of mutations in *MYD88* seen here in OAML is consistent with previously described *MYD88* mutations, as 8/12 (67%) of patients with *MYD88* mutations in this cohort exhibit the Leu265Pro mutation.

Mutations in the apoptosis regulator TP53 were detected in five cases, whereas *FAS* was not mutated in any of the cases. Thus, *TP53* is recurrently mutated in OAML, although at a relatively low frequency.

**Figure 2:**
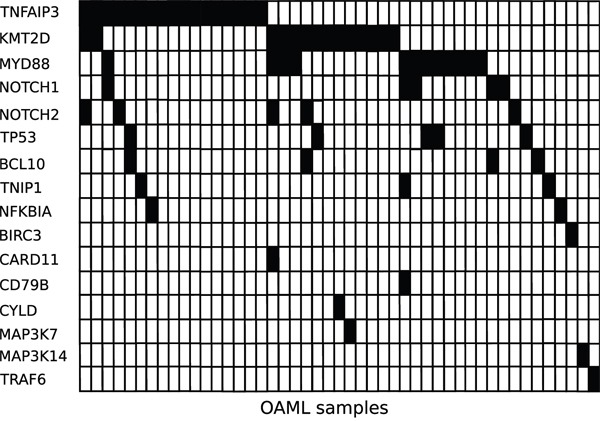
Distribution of mutated genes in the OAML Mutated genes are indicated as black fields for the 47 OAML that carried at least one mutated gene.

Figure 2 depicts the distribution of the mutated genes in the patient cohort. When considering the three most frequently mutated genes, which were each mutated in at least twelve cases, for a contingency analysis, there was a significant negative association observed between *TNFAIP3* and *MYD88* (p=0.034; Fisher' s exact test, two-sided).

### Newly identified recurrent mutations in *NOTCH1*, *NOTCH2*, and *KMT2D* in OAML

**Figure 3:**
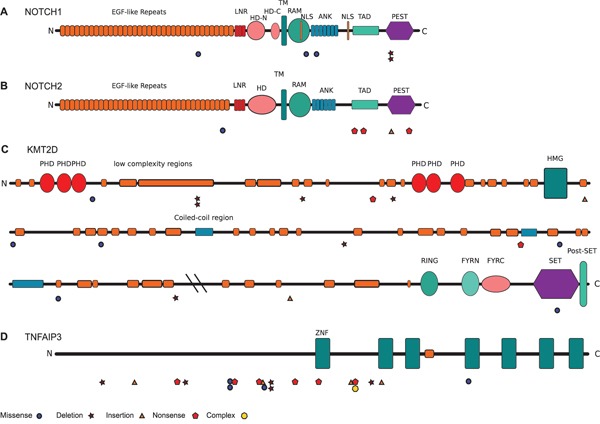
Pattern and distribution of mutations in *NOTCH1, NOTCH2, KMT2D*, and *TNFAIP3* Depicted are all mutations per gene. **A.** NOTCH1, **B.** NOTCH2, **C.** KMT2D, **D.** TNFAIP3. Abbreviations: ANK ankyrin; EGF epidermal growth factor; FYRC F/Y-rich, C-terminal; FYRN F/Y-rich, N-terminal; HD-C heterodimerization domain, c-terminal; HD-N heterodimerization domain, N-terminal; HMG high mobility group; LNR Lin12/NOTCH repat; NLS nuclear localization signal; PEST proline-glutamate-serine-threonine rich; PHD plant homeodomain; RAM retinole binding associated molecule; SET Su(var)3-9 - Enhancer of zeste' – Trithorax; TAD trans activating; TM transmembrane; ZNF zink finger.

Five of the 63 patients (8%) harbored mutations in the *NOTCH1* gene. The *NOTCH1* mutations were located in several exons which encode protein domains including the functionally important HD and PEST domains. Two frameshift mutations caused by deletions were detected in exon 34, which encodes the inhibitory PEST domain (Figure 3a). We detected recurrent *NOTCH2* mutations in 8% of cases. Most of these mutations were located in exons encoding for intracellular protein domains, namely the TAD and the PEST domains (Figure 3b). Importantly, the latter two domains were affected by nonsense or frameshift mutations. Mutations in *KMT2D* were identified in 22% of cases and were distributed along the whole length of the gene (Figure 3c). Numerous nonsense mutations and insertion or deletions, leading to frameshift mutations, occurred, most likely impairing protein function. Moreover, one missense mutation was located in the functionally relevant Su(var)3-9 - Enhancer of zeste' – Trithorax (SET) domain.

### Clonality versus subclonality of mutations

**Figure 4:**
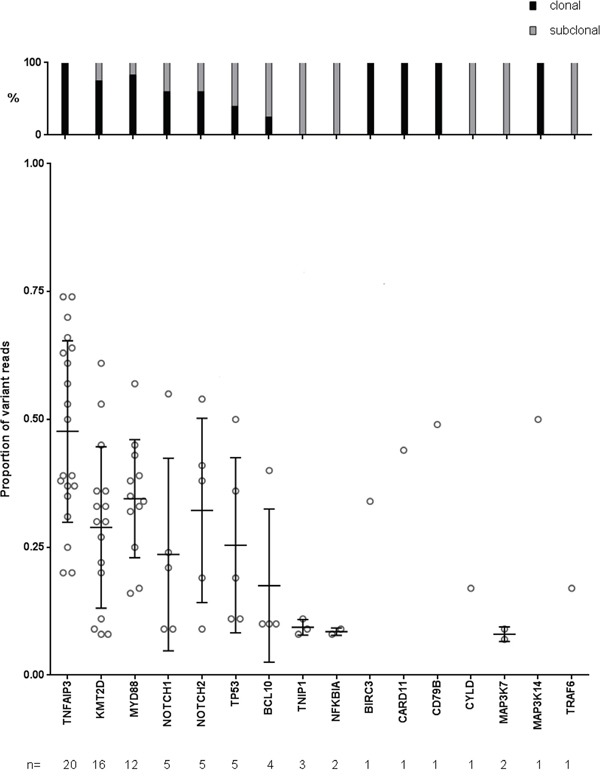
Variant allele frequencies of the 16 mutated genes Variant allele frequencies below 20% are considered as subclonal. Depicted are all mutations per gene. Bars depict mean and standard deviation.

In a targeted sequencing approach with DNA from whole tissue sections, it is often difficult to unequivocally determine whether a mutation is clonal or subclonal in the lymphoma cells. This is mainly due to the difficulty to determine the tumor cell content in the biopsy, the lack of knowledge about aneuploidy of chromosomes with mutations, and potential variation in the efficiency to amplify the wildtype and the mutated allele. In principle, one would expect, for example, in a case with 80% tumor cell content for a clonal heterozygous mutation a variant allele frequency (VAF) of about 40%. The mutations we detected in the OAML (selected to have at least 70% tumor cell content, according to histopathological estimation) showed a wide range of VAF (Figure 4). *TNFAIP3* had the highest average VAF of about 48%, indicating that mutations in this gene are mostly clonal. For cases harboring *TNFAIP3* mutations with VAF above 50%, it is likely that in these instances either the second allele is deleted in the tumor cells, or that we observe here uniparental disomy of the mutated allele. When setting a cut-off of <20% VAF for mutations that are very likely subclonal, it is evident that a number of genes analyzed harbor mostly if not always subclonal mutations. This includes *NOTCH1*, *TNIP1*, *MAP3K7*, *BCL10*, and *NFKBIA* (Figure 4). The fraction of subclonal mutations might well be considerably higher, taking into account the difficulty to quantify the tumor cell fraction based on histopathological evaluation and the technical variation inherent in the experiment.

### Validation of mutations

As we studied formalin-fixed paraffin embedded (FFPE) material, which often causes higher rates of false positive mutations in sequence analyses than good quality DNA, and as a substantial number of mutations were detected with relatively low VAF, it was important to clarify the reliability of our mutation analysis. To this end, we selected 33 mutations in 11 genes for 22 patients, including mutations with high as well as low VAF as well as low and high coverage for independent analysis by specific PCR and Sanger sequencing. The amplicons were either directly sequenced, or for mutations with low VAF they were first cloned into plasmids, and then 19-30 plasmids per PCR product were sequenced. This analysis revealed that none of 13 candidate mutations with a coverage below 100 was confirmed. However, for candidate mutations with a coverage above 100, 19 of 21 mutations were validated, independent of variant allele frequency (Supplementary Tables S2 and S3). Notably, the only two exchanges not validated among positions with more than 100 reads were an identical 1 bp deletion in Notch 1 (position 139390945, GRCh37). Consequently, we eliminated also further instances with the same deletion from the analysis.

A further validation was provided by the comparative amplicon sequencing analysis from DNA of tumor cell suspensions to the corresponding FFPE material of five patients. This analysis revealed that 8/8 mutations were identified in both of the paired samples (Supplementary Table S4). Moreover, for two cases with a primary biopsy and a relapse, the same two mutations were detected in both samples (not shown). Finally, we also sequenced DNA of FFPE material from biopsies diagnosed as reactive lymphoid hyperplasia of the ocular adnexa, and did not identify any mutation in these samples (data not shown). Thus, the reliability of the mutations reported here is well confirmed by the validation analyses.

We could not validate the somatic origin of the mutations that we describe here, as non-tumor DNA was not available from the patients. However, we are convinced that at least the vast majority of the mutations are indeed somatic, for the following reasons: i) We carefully filtered the mutations against known polymorphisms, ii) the recurrent Leu265Pro exchange in *MYD88* is well known from numerous studies of other lymphomas as a somatic mutation [22, 32, 33], iii) the location and pattern of several mutations described here (e.g. in *NOTCH1* and *NOTCH2*) very well fit to the locations and patterns known for these genes from studies of other B-NHL [25, 26, 29, 34], in which the somatic origin of these mutations were validated as somatic in origin, and iv) a substantial fraction of mutations described here were detected with VAF below 50%, which does not fit to a polymorphism, which should appear with a VAF of about 50%, independent of tumor cell content in the biopsy.

### Potential clinical impact of mutations

**Figure 5:**
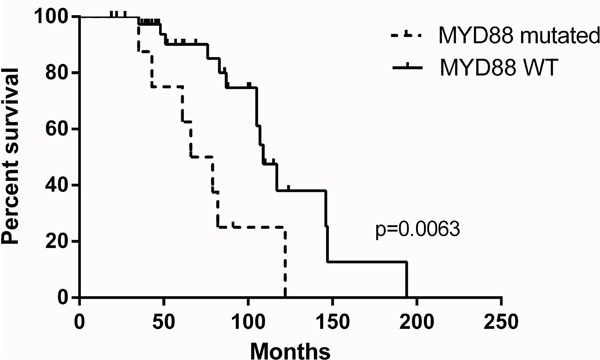
Kaplan-Meier estimates for disease-free survival regarding *MYD88* mutation status in the OAML patient cohort (n=63 patients) Note that the association of disease-free survival with MYD88 mutation status is still significant (p=0.0152) even if only the 54 patients with stage I disease, who all received radiotherapy, are considered (not shown).

We investigated whether the presence or absence of the most prevalent mutations were associated with the clinical course of the patients in this cohort. To this end we performed Kaplan-Meier analyses for the most frequently mutated genes using disease-free survival (DFS) as readout. Patients harboring a *MYD88* mutation in their lymphoma exhibited a significantly shorter DFS as compared to patients with wild type MYD88 (Figure 5, p=0.006). The occurrence of MYD88 mutations was not associated with other clinical characteristics, such as localization, Ann Arbor stage or treatment. Mutations in *NOTCH1, TNFAIP3, KMT2D*, or *NOTCH*2 did not appear to affect the clinical outcome of the patients (data not shown).

## DISCUSSION

The genetic lesions involved in the pathogenesis of OAML are still largely unknown. Chromosomal translocations involving the genes *BCL10* or *MALT1*, which are frequent in other types of MALT lymphomas, are rare in OAML [17, 19, 35, 36]. Several studies reported recurrent mutations in *TNFAIP3*, *MYD88* and *BCL10* in OAML [19, 20, 22, 23], and a few mutations were also found in *TNIP1*, *TNIP2*, *CARD11* and *BIRC3* [19, 22]. All of these genes are components of the NF-κB pathway, indicating a major role of its genetic deregulation in OAML, similar to other MALT lymphomas [15]. This prompted us to perform a targeted sequence analysis of 19 genes of the NF-κB pathway in a large cohort of OAML. We complemented the analysis by inclusion of two main apoptosis regulators, i.e. FAS and TP53, which are known to be mutated in some B-NHL, as well as the three genes *NOTCH1*, *NOTCH2* and *KMT2D* that were recently identified as recurrently mutated in several types of B-NHL [24–31], but were not studied yet in OAML.

We validated frequent mutations in *TNFAIP3* and *MYD88* in OAML, as well as rare mutations in *BCL10*, *TNIP1*, *CARD11*, and *BIRC3*. The frequency of *TNFAIP3* mutations seen in our analysis (27% of cases; pattern shown in Figure 3d) is similar to prior studies [19, 22]. For *MYD88*, the mutation frequency in our cohort (19%) is considerably higher than in most other studies, which reported 5-10% of OAML with such mutations (or in one study 0%) [17, 22, 37, 38], but in line with a recent publication [39]. This might reflect regional differences, as four of the five prior studies on *MYD88* mutations in OAML involved patients from Asia or North America. Another explanation might be that we applied targeted sequencing, so that also subclonal mutations were identified, which might have been missed in the other studies using conventional Sanger sequencing. However, the majority of *MYD88* mutations were clonal in the cases analzyed here.

Our analysis revealed several additional mutated members of the NF-κB pathway that were not previously known to be mutated in OAML. These are the genes MAP3K7 (2%), MAP3K14 (2%), CYLD (2%), CD79B (2%), TRAF6 (2%), and NFKBIA (3%). Thus, there are many more factors of NF-κB signaling mutated in OAML than known from earlier analyses, and overall 60% of the 63 cases studied here carried non-synonymous mutations in at least one of these genes. Considering that gene deletions and chromosomal translocations were not investigated here, and that we did not include all genes that play a role in NF-κB signaling, the true frequency of OAML with genetic lesions affecting the NF-κB pathway might even be higher. Most of the mutations identified here as well as the known translocations involving MALT1 or BCL10 affect the canonical NF-κB pathway. However, one case harbored a mutation in MAP3K14, the key activating kinase of the non-canonical pathway, and the genes *BIRC3* and *CYLD*, which were both mutated in one case each, play a role in both NF-κB pathways, so that in rare instances, also the non-canonical NF-κB pathway is affected by mutations in OAML.

*NOTCH1* is recurrently affected by activating mutations in about 10-20% of chronic lymphocytic leukemias and mantle cell lymphomas, and in a lower fraction of diffuse large B cell lymphomas, follicular lymphomas and splenic marginal zone lymphomas [25, 26, 29, 34, 40]. We identified here non-synonymous *NOTCH1* mutations in 8% of OAML (Figure 1). Moreover, *NOTCH2*, which is mutated in 20-25% of splenic marginal zone lymphomas, and less than 10% of diffuse large B cell lymphomas, was mutated in 8% of the OAML (Figure 1) [26, 40]. The pattern of mutations in the two genes in the OAML is similar to the pattern described for the other B-NHL, with a clustering of the mutations in the HD and PEST domains of NOTCH1, and downstream of the Ankyrin repeats in the intracellular domain of NOTCH2 (Figure 3). For both genes, these types of mutations cause a gain of function, as the inhibitory PEST domains are removed or otherwise inactivated. The bidirectional link between NOTCH1 and NF-κB is well known, indicating a promotion of tumor cells upon NOTCH1 activation [41, 42]. Notably, two of five *NOTCH1* mutations in the OAML were subclonal (Figure 4). These mutations are, nevertheless, likely of importance, because the pathogenetic relevance of subclonal mutations is well established for other B cell malignancies, in particular chronic lymphocytic leukemia [43], and there are numerous examples where a particular feature of a tumor subclone can lead to a selective advantage for the whole tumor [44]. Indeed, also in chronic lymphocytic leukemia, *NOTCH1* mutations are subclonal in about 50% of cases with such mutations [45].

The frequency of *NOTCH2* mutations in OAML is similar to other marginal zone lymphomas. There are yet no comprehensive data about *NOTCH1* mutations in other nodal or extranodal marginal zone lymphomas. In two prior studies of 8 and 17 primary OAML for *NOTCH1* exon 34 mutations not a single mutation was found, perhaps due to the fact that subclonal mutations, as identified in *NOTCH1*, might not be easily detectable in the sequencing approaches used in those studies [39, 46].

*KMT2D* encodes a histone methyltransferase that targets the Lys-4 position of histone H3. It is mutated in about 90% of follicular lymphomas, 30% of diffuse large B cell lymphomas, 15% of splenic marginal zone lymphomas, as well as in 10-15% of mantle cell lymphomas [24, 27, 28, 30, 31]. In our cohort, *KMT2D* mutations were the second most frequently found aberrations, affecting 22% of cases. Thus, we identified here OAML as a further B cell lymphoma with highly recurrent *KMT2D* mutations and the first epigenetic regulator involved in the pathogenesis of OAML.

Finally, we aimed to investigate the potential prognostic impact of our findings by comparing the clinical outcome of patient subgroups defined by the presence or absence of the most common mutations using DFS as a clinical read out. Importantly, we found that patients harboring a *MYD88* mutation in their tumor cell clone exhibited a significantly shorter DFS as compared to individuals without this genetic abnormality. This finding is reminiscent of two previous studies showing that activating *MYD88* mutations may serve as an adverse prognostic marker in aggressive B cell lymphomas [47, 48]. However, our results regarding the prognostic value of MYD88 in OAML are limited by the relatively small number of patients analysed and the retrospective nature of our study. Thus, these encouraging findings need to be validated in an independent patient cohort and ideally in the context of a prospective trial of homogenously treated patients with OAML.

Taken together, we identified several additional components of NF-κB signaling with recurrent genetic lesions in OAML, with an overall fraction of more than 60% of cases with mutations in at least one NF-κB regulator. Thus, genetic lesions in the NF-κB pathway play an even more critical role in the pathogenesis of OAML than implied from prior studies. Moreover, recurrent mutations in *NOTCH1* and *NOTCH2* were revealed, and *KMT2D* mutations are shown here to be highly recurrent in OAML. Finally, we provide first indication that *MYD88* mutations might be of prognostic relevance.

## MATERIALS AND METHODS

### Patients

Patient samples were obtained from archived material of the Universities of Duisburg-Essen and Kiel, and the Kliniken Essen-Mitte. Seventy-six FFPE samples were initially selected for analysis with the approval of the ethical review committees of the Universities of Kiel and Duisburg-Essen. Clinical data of 63 patients, for whom evaluable sequences were obtained, are summarized in Table 1. For three of these 63 patients, additional cell suspensions obtained at the same time points as the corresponding FFPE material were collected. For three further patients, additional FFPE samples from a later time point were available. Two of these were used for validation studies. However, the third case was not informative for validation studies, because the relapse expressed Igλ light chains, whereas the primary OAML showed Igκ expression, indicating that the relapse and the primary OAML are separate lymphoma clones. Patients were diagnosed between 2005 and 2012 in accordance with the WHO 2008 classification [49]. The diagnosis was based on morphologic evaluation of immunohistochemical stainings on sections with antibodies against CD3, CD5, CD10, CD20, CD23, CD43, CD11c, CD79a, BCL2, immunoglobulin kappa and lambda light chains, cyclin D1 and Ki-67, often complemented by analysis for clonal IgH rearrangements (not shown). Only patients with primary OAML were included in this study. No patient had a high grade component. Clinical information was available for most patients. For 11 of the 12 patients with *MYD88* mutations, the clinical data clearly excluded a differential diagnosis of lymphoplasmacytic lymphoma, the remaining case was one of two *MYD88*-mutated patients with paraproteinemia. The second case with paraproteinemia had a localized disease (Ann Arbor stage IEA) and no histologic evidence for plasmacytic differentiation. Standard clinical criteria were used for the initiation of therapy.

### Sample collection and DNA isolation

Sections from FFPE tissue samples were obtained from archived material. FFPE material contained at least 70% tumor cells according to immunohistochemical evaluation. DNA was extracted from up to eight 10 μm thick sections per sample using the QIAamp DNA FFPE Tissue Kit (Qiagen, Hilden, Germany). DNA concentrations were measured with Qubit Fluorometer 2.0 (Life Technologies, Darmstadt, Germany). Sufficient amounts of DNA for further analysis were isolated from 69 of 76 archived FFPE samples. DNA of cell suspensions from five cases was extracted with the QIAamp Blood mini kit (Qiagen). All preparations were carried out according to the manufacturer's guidelines. Between 47 ng and 3.7 μg DNA were isolated from the samples. Two patients were excluded from further analysis due to poor DNA quality.

### Multiplex PCR and sequencing

For multiplex PCR two customized Ion AmpliSeq primer pools for a total of 1255 amplicons were used (Life Technologies, Darmstadt, Germany). Primer sequences are given in Supplementary Table S5. The DNA input per reaction was 10 ng. Multiplex PCR was performed for 24 target genes (Table 2), most of which are involved in NF-κB signaling. The PCR program consisted of 99°C for 2 minutes, followed by 23 cycles of 99°C for 15 seconds and 60°C for 4 minutes. Libraries were prepared with the NEBNext Ultra DNA Library kit (NEB, Ipswich, UK) for sequencing on the Illumina platform. Purified libraries were quantified with the KAPA library quantification kit (Peqlab, Erlangen, Germany). Library size and quality was determined with the Agilent Bioanalyzer high sensitivity DNA assay (Agilent, Santa Clara, USA). Sequencing was performed on an Illumina MiSeq apparatus with 150 bp paired end reads.

### FFPE DNA sequencing analysis and data processing

Sequence reads were mapped to the human genome reference assembly GRCh37 (hg19). Alignment was done using Bowtie 2 [50]. For variant calling, Avadis NGS Version 1.2 was used [51]. Settings for filtering were as follows: the phred quality score was set >30. Positions with a coverage below 100 for a given sample were not considered. In general, variants were accepted only if occurring in at least 20% of reads, variants with frequencies between 5-20% were analyzed manually using IGV [52]. Mutations occurring at the end or beginning of an amplicon near to the primer, mutations, which only occur in reads of one amplicon and not in the reads of the overlapping adjacent amplicon and amplicons, which are mapped to the wrong reference sequence (e.g. cases with homologous sequences and many mismatches) were excluded as unreliable. Known SNPs (db137) were excluded. Non-synonymous substitutions were considered to be damaging when three of five prediction tools (SIFT, PolyPhen2 HDIV, PolyPhen2 HVAR, LRT, MutationTaster) [53–56], which predict a possible impact of an amino acid substitution on protein structure and function, gave similar results. Sequences were submitted to the SRA database under accession number SRP076171.

### Amplification and sequence analysis of mutations

To validate the candidate mutations detected by DNA sequencing, we selected several mutated positions with ≥20% variant calls (Supplementary Table S2). After PCR, amplicons were analyzed by Sanger sequencing (ABI3130 Genetic Analyzer; Applied Biosystems, Life Technologies). Primer sequences are available from the authors upon request. For mutations with <20% of variant calls, amplicons were cloned. PCR products were ligated into the pGEM T-easy vector (Promega, Madison, WI, USA). After transformation in XL1-Blue (Stratagene, Waldbronn, Germany) JM109 (Promega) competent cells, plasmids were isolated (mi-Plasmid Miniprep Kit, Metabion, Planegg/Steinkirchen, Germany) and individual clones were sequenced by Sanger sequencing. Sequences of PCR products were compared to the corresponding germ line sequences with SeqScape v2.5 software (Applied Biosystems).

## SUPPLEMENTARY TABLES






