# Supplementary material for: Recurrent mutations in NF-κB pathway components, KMT2D, and NOTCH1/2 in ocular adnexal MALT-type marginal zone lymphomas
Source: Oncotarget. 2016 Aug 23;7(38):62627–39. doi: 10.18632/oncotarget.11548 (PMC5308752; doi:10.18632/oncotarget.11548)
Supplement: Supplementary file 1 [file oncotarget-07-62627-s001.pdf]

Recurrent mutations in NF-κB pathway components, *KMT2D,* and *NOTCH1/2* in ocular adnexal MALT-type marginal zone lymphomas

SUPPLEMENTARY TABLES

Supplementary Table S1:

See Supplementary File 1

Supplementary Table S2: Mutations validated by direct Sanger sequencing of PCR products

Patient ID	Gene	Chr	Position (hg19)	Transcript	Predicted consequence	CDS position	Predicted protein change	VAF	Mutation validated
2; 18; 21	*MYD88*	3	38,182,641	NM_002468	NON_ SYNONYMOUS_ CODING	c.978T>C	p.Leu265Pro	0.45; 0.42; 0.35	Yes; Yes; Yes
61	*TNFAIP3*	6	138,198,218	NM_006290	STOP_GAINED	c.1133C>T	p.Arg271X	0.45	Yes
56	*TNFAIP3*	6	138,196,885	NM_006290	STOP_GAINED	c.869C>T	p.Arg183X	0.20	Yes
3	*KMT2D (MLL2)*	12	49,442,925	NM_003482	FRAMESHIFT_ CODING	c.3982CG>C		0.36	Yes
1	*KMT2D (MLL2)*	12	49,426,316	NM_003482	NON_ SYNONYMOUS_ CODING	c.12172T>C	p.Met4058Val	0.53	Yes
39	*NOTCH2*	1	120,458,447	NM_024408	FRAMESHIFT_ CODING	c.7195G>GCTCC		0.37	Yes
45	*TP53*	17	7,577,517	NM_001126117	NON_ SYNONYMOUS_ CODING	c.646A>G	p.Ile123Thr	0.35	Yes
2	*TP53*	17	7,578,271	NM_001126117	NON_ SYNONYMOUS_ CODING	c.460T>A	p.His61Leu	0.19	Yes
20	*MAP3K14*	17	43,351,496	NM_003954	EXONIC		p.Val674Ala	0.49	Yes
34	*CARD11*	7	2,979,559	NM_032415	NON_ SYNONYMOUS_ CODING	c.1022C>T	p.Asp230Asn	0.43	Yes
14	*BCL10*	1	85,733,589-85,733,591	NM_003921	FRAMESHIFT_ CODING	c.973-974ACT>A		0.39	Yes
14	*KMT2D*	12	49,427,350	NM_003482	STOP_GAINED	c.11138G>C	p.Ser3713X	0.30	Yes
27	*NOTCH1*	9	139,390,944	NM_017617	FRAMESHIFT_ CODING	c.7246TG>T		0.16	No
31	*NOTCH1*	9	139,390,944	NM_017617	FRAMESHIFT_ CODING	c.7246TG>T		0.12	No

CDS = Coding sequence, Chr = chromosome, VAF= variant allele frequency.

Supplementary Table S3: Mutations validated by cloning and Sanger sequencing

Patient ID	Gene	Chr	Position (hg19)	Transcript	Predicted consequence	CDS position	Predicted protein change	VAF	Sequences with mutation/total sequences*
25	*BCL10*	1	85,742,025	NM_003921	NON_ SYNONYMOUS_ CODING	c.563G>C	p.Thr4Ser	0.09	1/23
28	*CYLD*	16	50,815,297	NM_015247	NON_ SYNONYMOUS_ CODING	c.2074T>G	p.Asn553Lys	0.17	3/21
25	*NOTCH1*	9	139,390,648	NM_017617	FRAMESHIFT_ CODING	c.7541-7542CAG>C		0.09	13/19
35	*NOTCH1*	9	139,390,648	NM_017617	FRAMESHIFT_ CODING	c.7541-7542CAG>C		0.09	13/21
L13	*TRAF6*	11	36,511,891	NM_145803	STOP_GAINED	c.1447T>A	p.Lys356X	0.16	4/23

CDS = Coding sequence, Chr = chromosome, VAF= variant allele frequency.
*The validation analysis indicated in two instances a higher VAF than the one calculated in the amplicon sequencing. This was observed at a position in NOTCH1 which is in one of two overlapping amplicons located at the end of the amplicon, in a region already covered by the PCR primer. During library preparation, primers were sometimes only partially removed, so that a fraction of the reads still contains parts of the primers, wich are "wildtype" at the mutated position. As the residual four to five bases of primers at the ends of reads could not be removed by the evaluation software, this causes for the respective mutated position many reads with wildtype sequence from the amplicon covering that position in the primer binding site. Importantly, this problem involves only a few mutations that we describe here. Thus, in rare instances we underestimate the VAF of mutated positions.

Supplementary Table S4: Mutations in corresponding FFPE samples and cell suspensions

Patient ID	Gene	Chr	Position (hg19)	Transcript	Predicted consequence	CDS position	Predicted protein change	VAF*	Presence of mutation
39; L2	*KMT2D*	12	49,431,953	NM_003482	FRAMESHIFT_CODING	c.9185CT>C		0.36; 0.32	confirmed
	KMT2D	12	49,427,265	NM_003482	NON_SYNONYMOUS_CODING	c.11220-11222TTGC>T		0.08; 0.57	confirmed
	NOTCH2	1	120,458,447	NM_024408	FRAMESHIFT_CODING	c.7195G>GCTCC		0.38; 0.38	confirmed
	TNFAIP3	6	138,198,315	NM_006290	STOP_GAINED	c.1230T>A	p.Lys303X	0.53; 0.39	confirmed
19; L4	*MYD88*	3	38,182,641	NM_002468	NON_SYNONYMOUS_CODING	c.978T>C	p.Leu265Pro	0.43; 0.43	confirmed
38; L1	*NOTCH1*	9	139,390,648	NM_017617	FRAMESHIFT_CODING	c.7541CAG>C		0.18; 0.02	confirmed
	TNFAIP3	6	138,197,133	NM_006290	FRAMESHIFT_CODING	c.958AC>A		0.74; 0.77	confirmed
	TNFAIP3	6	138,199,910	NM_006290	FRAMESHIFT_CODING	c.1650A>AT		0.57; 0.49	confirmed

*The first number refers to the VAF in the FFPE material, the second number to the VAF determined for the cell suspension.

Supplementary Table S5:

See Supplementary File 2
